# Cardiac iron overload promotes cardiac injury in patients with severe COVID-19

**DOI:** 10.1007/s15010-021-01722-6

**Published:** 2021-10-20

**Authors:** Maria J. Baier, Stefan Wagner, Julian Hupf, Katja Evert, Matthias Evert, Samuel Sossalla, Carsten Jungbauer, Lars S. Maier, Stefan Neef, Julian Mustroph

**Affiliations:** 1 Department of Internal Medicine II (Cardiology), University Heart Center Regensburg, University Hospital Regensburg, Franz‐Josef‐Strauß‐Allee 11, 93053 Regensburg, Germany; 2 Emergency Department, University Hospital Regensburg, Franz-Josef-Strauß-Allee 11, 93053 Regensburg, Germany; 3 Institute of Pathology, University of Regensburg, Universitätsstraße 31, 93053 Regensburg, Germany

During the current SARS-CoV-2-pandemic, involvement of multiple organs in addition to the respiratory tract has been observed. Cardiac manifestations, such as acute heart failure, arrhythmias or acute coronary syndrome, are known complications in severe disease courses [1].

Multiple studies have reported hyperferritinemia in patients with COVID-19 infection and suggested dysregulated iron metabolism in severe COVID-19 disease courses [2]. Recently, iron deficiency was linked to significantly longer hospital stay and the need for ICU admission and mechanical ventilation [3, 4]. However, no direct correlation between cardiac iron homeostasis and severity of COVID-19 infection has been shown yet.

Dysregulated iron homeostasis is accompanied by ferroptosis, a newly identified type of controlled cell death, which is triggered by intracellular iron accumulation, decreased iron storage capacity and glutathione depletion, leading to excessive reactive oxygen species (ROS) formation, lipid peroxidation and, consequently, cell death [5].

In this study, we investigated iron metabolism in left ventricular myocardium of patients who had died due to severe COVID-19 and assessed cardiac iron content as well as ferroptosis pathways in these patients. To our knowledge, this is the first study showing a direct relationship between COVID-19 infection, cardiac iron overload, and cardiac injury in the myocardium.

All investigations conform to the Declaration of Helsinki. Informed consent for autopsies involving scientific investigations had been obtained from relatives of all deceased patients, which was performed in accordance with institutional and ethics guidelines. We obtained left ventricular cardiac tissue from autopsies of patients who had died of COVID‐19. In all these patients, infection was verified by PCR from respiratory material, and all patients had symptomatic disease, which was determined to be the cause of death. Because control tissue is extremely rare, we used a combination of left ventricular tissue from patients whose hearts were intended for heart donation that ultimately could not be performed (“NF” = non-failing, for iron assay and qPCR), and from patients who had died from sepsis or other respiratory infections (“SEPSIS”, for qPCR and immunofluorescence). Clinical characteristics of the “COVID-19” and “SEPSIS” patient groups can be found in Table 1. No clinical data is available regarding the non-failing group.

**Table 1:** Baseline characteristics of CTRL and COVID-19 patients

	SEPSIS (*n* = 9)	COVID-19 (*n* = 9)	*p* value
Age	57.67 ± 4.97	61.44 ± 3.06	0.53
Sex, % male (*n*)	77.78 (7)	55.56 (5)	0.32
SARS-CoV-2 infection confirmed by PCR: % (*n*)	0 (0)	100 (9)	* < 0.0001
Intubation: % (*n*)	77.78 (7)	100 (9)	0.13
VV- or VA-ECMO: % (*n*)	11.11 (1)	55.56 (5)	*0.04
BMI	24.98 ± 2.02	33.28 ± 2.66	*0.02
Diabetes, % (*n*)	22.22 (2)	22.22 (2)	> 0.99
Arterial Hypertension, % (*n*)	33.33 (3)	66.67 (6)	0.16
Coronary artery disease, % (*n*)	11.11 (1)	22.22 (2)	0.53
Heart failure, % (*n*)	22.22 (2)	11.11 (1)	0.53
End-stage renal disease, % (*n*)	22.22 (2)	88.89 (8)	*0.004
Ferritin^a^	2747 ± 2162 (*n* = 6)	7455 ± 4305 (*n* = 7)	0.23
Serum iron^a^	125.8 ± 57.43 (*n* = 4)	26 (*n* = 1)	0.49
Hemoglobin	8.96 ± 0.76	10.08 ± 1.22	0.43

*PCR* polymerase chain reaction, *vv* veno-venous, *va* veno-arterial, *ECMO* extracorporeal membrane oxygenation

^a^Number of values available differs from group number

* *p* value <0.05

We assessed the iron content in left ventricular myocardium using an iron assay kit (ab83366, abcam), which was performed according to manufacturer’s instructions. Briefly, 10 mg of left ventricular tissue was homogenized in 500 µl Iron Assay Buffer (+ 10% complete protease inhibitor cocktail, Roche diagnostics). After centrifugation (16,000×*g*, 10 min) the supernatant was collected and incubated with Iron Reducer (37 °C, 30 min) to assess total iron. Afterwards, 100 µl of standard (0, 2, 4, 6, 8, 10 nmol/well) and sample probes (in duplicates) were incubated with 100 µl Iron Probe for 60 min at 37 °C. Absorption was measured at 593 nm. Total iron concentration in the samples was calculated using standard curve and sample volume.

Western Blot analysis was performed as described previously [6]. Briefly, LV myocardium was mechanically homogenized (using a stainless steel pestle) in Tris buffer containing (in mmol/L): 50 Tris–HCl, 200 NaCl, 20 NaF, 1 Na_3_VO_4_, 1 DTT [pH 7.4] and protease inhibitor cocktail. Protein concentration was determined by BCA assay. Proteins were denaturated for 30 min at 37 °C in 2% β-mercaptoethanol. Proteins were separated on 8% sodium dodecyl sulfate polyacrylamide gels, then transferred to a nitrocellulose membrane and incubated with the primary antibodies in 5% milk in TBS-T at 4 °C overnight: anti-FTH (ab65080, abcam, 1:1000), anti-FTL (ab69090, abcam, 1:1000) and as housekeeper anti-GAPDH (G8795, Sigma-Aldrich, 1:10,000). Secondary antibodies were HRP-conjugated donkey anti-rabbit and sheep anti-mouse IgG (NA934 and NA931, GE Healthcare, 1:10,000) that were incubated for 1 h at room temperature. For chemiluminescent detection, WesternBright^TM^ ECL HRP substrate (K-12045-D50, advansta) was used. Protein expression was normalized to GAPDH expression using ImageJ to determine mean densitometry.

In addition, RNA was extracted from left ventricular tissue using trichloromethane–chloroform solution and isopropanol solution. RNA was purified using the RNeasy Plus Mini Kit (Qiagen) and transcribed to cDNA. Quantitative analysis was performed using the respective primer (FTH: Hs01694011_s1, FTL: Hs00830226_gH, GPX4: Hs00989766_g1, HMOX-1: Hs01110250_m1, Applied Biosystems) on a TaqMan apparatus (Applied Biosystems), and expression was normalized to β‐actin (Hs00357333_g1, Applied Biosystems).

For detection of 4-HNE, a marker of oxidative stress, paraffin embedded hearts were cut in 4 µm thick sections and fixed on slides. Before staining, sections were deparaffinized and rehydrated (2 × 10 min in xylol, 2 × 5 min in 100% EtOH, 2 × 5 min in 96% EtOH, 2 × 5 min in 70% EtOH, 5 min in PBS). Antigen retrieval was performed by 30 s boiling at 121 °C in Antigen Unmasking Solution (H-3300-250, Vector Laboratories, 1:100 in dH_2_O). After blocking with SuperBlock™ Blocking Buffer (37515, Thermo Fisher Scientific) for 30 min, heart sections were stained with Wheat Germ Agglutinin (WGA), Alexa Fluor™ 594 Conjugate (W11262, Thermo Fisher Scientific, 1:300 in PBS, marker for cell membrane) and Anti-4 Hydroxynonenal antibody (4-HNE) (ab48506, abcam, 1:200 in PBS) antibody overnight (4 °C). Donkey anti-mouse IgG Alexa Fluor™ 568 (A10037, Invitrogen, 1:500 in PBS) was used as secondary antibody for 4-HNE (1 h at room temperature). Staining with DAPI (33342, Molecular Probes, 1:50,000 in PBS, 2 min) was done to visualize the nuclei. To quench autofluorescence, sections were incubated with Vector^®^ TrueVIEW^®^ Autofluorescence Quenching Kit (SP-8400-15, Vector Laboratories) for 2 min. For analysis, 40 images per section were recorded with a Zeiss Observer.Z1 microscopy (20x, Carl Zeiss Microscopy GmbH) and percent of 4-HNE stained area of total area was calculated.

For statistical testing, normality was tested using the Kolmogorov–Smirnov test. For data with normal distribution, a Student’s *t* test, one-way ANOVA with Šídák’s multiple comparisons test (for equal SD) or Brown-Forsythe/Welch ANOVA test with two-stage linear step-up procedure of Benjamini, Krieger and Yekutieli (no equal SD) was performed. For data, for which normality could not be assumed, Mann–Whitney test or Kruskal–Wallis with Dunn’s multiple comparisons test was performed. For correlation analysis, correlation was tested using linear regression. The significance level was taken to 5% (two‐sided *p*). Data are presented as mean ± SEM. Analyses and graphs were generated using GraphPad Prism 9 Statistical Software (GraphPad, San Diego, CA, USA).

Iron concentration in left ventricular myocardium of patients who had died due to COVID-19 was significantly elevated compared to tissue from the NF (non-failing) group (Fig. 1A, NF: *n* = 6, COVID-19: *n* = 7). To determine the cause of increased iron accumulation in the heart of COVID-19 patients, we analyzed the expression of genes involved in iron metabolism.

**Fig. 1:**
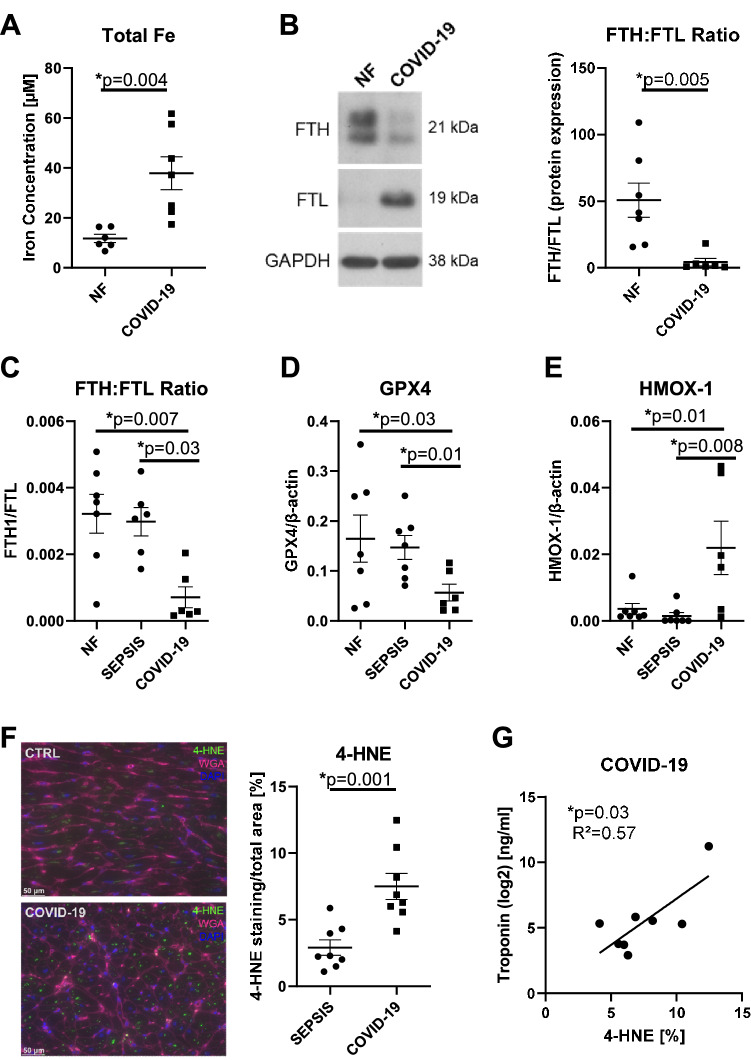
Iron metabolism in left ventricular (LV) myocardium of deceased COVID‐19 patients. **A** In the LV myocardium of deceased COVID‐19 patients, iron concentration is significantly increased compared to the non-failing group (NF; *p* = 0.004, *t* test, *n* = 6–7). **B** In LV myocardium of deceased COVID‐19 patients, protein expression of ferritin heavy chain (FTH) to ferritin light chain (FTL) is significantly decreased compared to NF group (*p* = 0.005, Mann–Whitney test, *n* = 6–7). **C** In LV myocardium of deceased COVID‐19 patients, ratio of ferritin heavy chain (FTH) to ferritin light chain (FTL) mRNA is significantly decreased compared to NF (*p* = 0.007) and SEPSIS group (*p* = 0.03, Kruskal–Wallis test, *n* = 6–7). **D** In LV myocardium of deceased COVID-19 patients, glutathione peroxidase 4 (GPX4) is decreased compared to NF (*p* = 0.03) and SEPSIS group (*p* = 0.01, Brown-Forsythe/Welch ANOVA test, *n* = 6–7). **E** In LV myocardium of deceased COVID‐19 patients, heme oxygenase-1 (HMOX-1) is significantly increased compared to NF (*p* = 0.01) and SEPSIS group (*p* = 0.008, one-way ANOVA test, *n* = 6–7). **F** In LV myocardium sections of deceased COVID-19 patients, 4-HNE staining, a marker for oxidative stress, is significantly enhanced compared to SEPSIS group (*p* = 0.001, *t* test, *n* = 8 each) (4-HNE: green, WGA: red, DAPI: blue). **G** 4-HNE staining and troponin levels are significantly correlated in COVID-19 patients (*p* = 0.03, R^2^ = 0.57, *n* = 8, linear regression)

Intracellular iron accumulation may be accompanied by decreased iron storage capacity, which can be determined by the ratio of heavy chain ferritin (FTH) to light chain ferritin (FTL). Because FTH differs from FTL in its ferroxidase activity, a decreased FTH:FTL ratio results in decreased intracellular storage capacity as well as an increased labile iron pool (LIP) size, which increases oxidative stress. Protein expression of FTH:FTL ratio was significantly decreased in patients with COVID-19 compared to NF group (Fig. 1B, NF: *n* = 7, COVID-19: *n* = 6). It could be argued, that ferritin expression might also be altered in patients with "classic" i.e. bacterial septic shock. However, the ratio of FTH to FTL gene expression in left ventricular myocardium of COVID-19 patients was significantly decreased compared with tissues from the NF group, but also compared with patients who had died of sepsis (SEPSIS) (Fig. 1C, NF: *n* = 7, SEPSIS: *n* = 6, COVID-19: *n* = 6).

Similarly, glutathione peroxidase 4 (GPX4), an enzyme converting reduced glutathione (GSH) to oxidized glutathione (GSSG), was decreased in patients with COVID-19 compared with the NF and SEPSIS groups (Fig. 1D, NF: *n* = 7, SEPSIS: *n* = 7, COVID-19: *n* = 6), which mechanistically should further increase the labile iron pool (LIP).

Moreover, the LIP can be enhanced by excessive activation of heme oxygenase-1 (HMOX-1) (non-canonical ferroptosis pathway). Heme oxygenase catalyzes the degradation of heme to CO, biliverdin and free iron. In this study, HMOX-1 was significantly upregulated in patients who died from COVID-19 compared with both the NF and SEPSIS groups (Fig. 1E, NF: *n* = 7, SEPSIS: *n* = 7, COVID-19: *n* = 6), suggesting that both canonical and non-canonical ferroptosis pathways are involved in COVID-19 associated cell death. The increased LIP can lead to an excessive induction of reactive oxygen species (ROS) in the myocardium of these patients.

To test whether increased ROS-production leads to oxidative damage of the cells, we used 4-HNE (4-Hydroxynonenal) staining as a sensitive marker for oxidative damage and lipid peroxidation. The percentage area of 4-HNE staining was significantly increased in left ventricular sections from patients with COVID-19 compared with sections from patients in the SEPSIS group (Fig. 1F, SEPSIS: *n* = 8, COVID-19: *n* = 8). To test whether oxidative stress following myocardial iron overload could lead to myocardial damage in COVID-19 patients, we performed a correlation analysis between the percentage area of 4-HNE staining and troponin before death of the COVID-19 patients. Interestingly, our analysis showed a significant correlation of 4-HNE staining and troponin levels (Fig. 1G, COVID-19: *n* = 8), which would be consistent with ferroptosis-induced myocardial injury in severe COVID-19.

The current study demonstrates that iron overload occurs in the myocardium during severe disease courses of COVID-19. Intracellular iron accumulation is accompanied by decreased iron storage capacity (FTH:FTL ratio), inhibition of detoxifying enzymes (GPX4) as well as activation of the ferroptosis pathway (HMOX-1), leading to increased myocardial oxidative stress with subsequent myocardial damage (Fig. 2). Notably, our data suggest a mechanistic difference in iron metabolism between severe COVID-19 disease and bacterial sepsis. However, it is unknown whether impaired iron homeostasis predisposes to severe COVID-19 or is a direct effect of the disease.

**Fig. 2:**
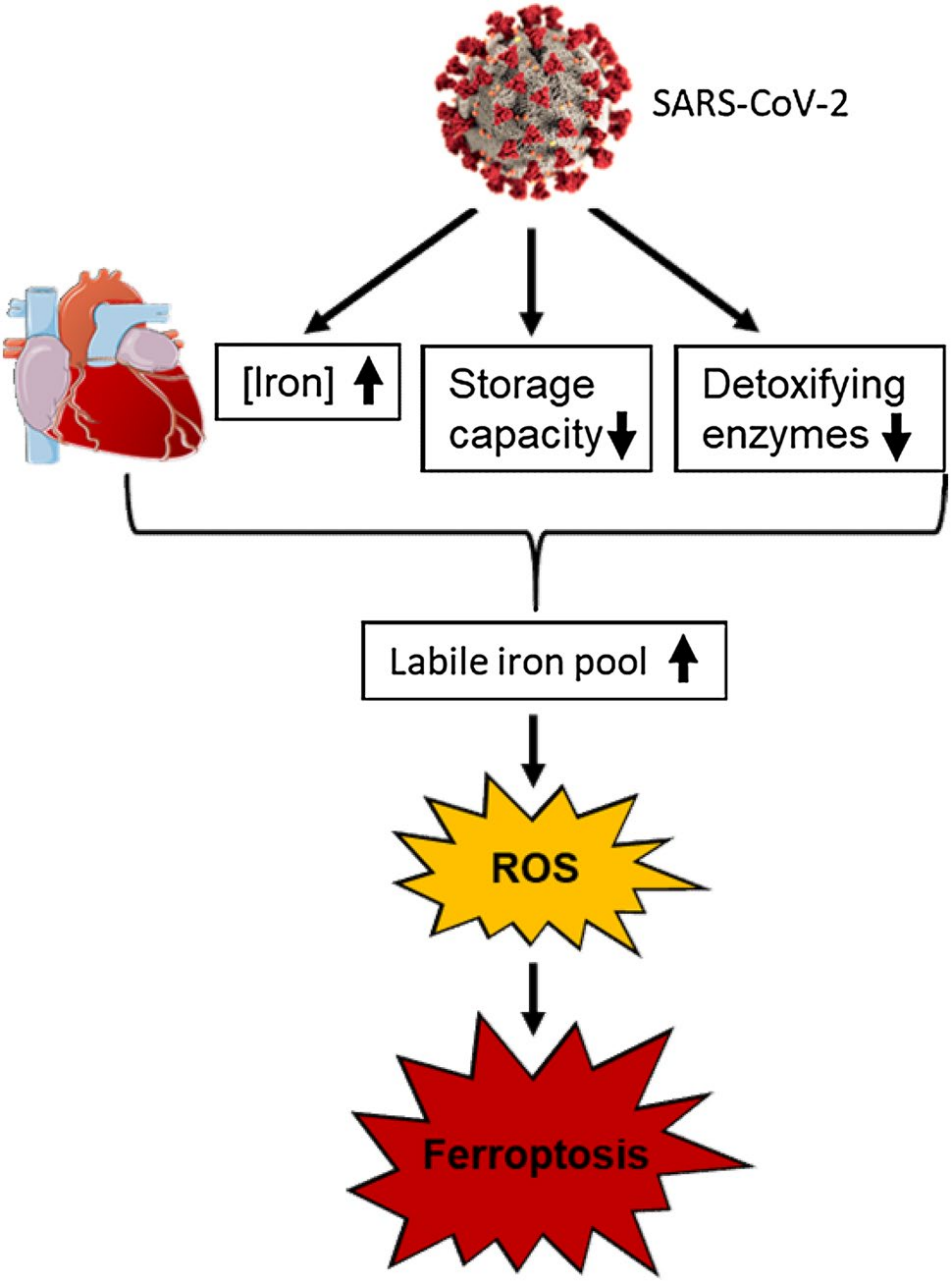
Graphical overview. In myocardium, SARS-CoV-2 infection increases iron concentration, decreases intracellular iron storage capacity and inhibits detoxifying enzymes, leading to excessive increase in the labile iron pool (LIP). As a result, reactive oxygen species (ROS) formation and lipid peroxidation occur, leading to ferroptosis. (SARS-CoV-2 figure by CDC/Alissa Eckert, MS; Dan Higgins, MAMS)

Since iron is also important for replication of viruses including coronaviruses, direct manipulation of iron metabolism by SARS-CoV-2 and dysregulated iron metabolism as a consequence of viral infection seems possible. This could also explain the heterogeneous clinical symptoms and multiple organ involvement in COVID-19, as ferroptosis has been shown to be involved in neurological diseases, renal damage, the circulatory system, and even immune system regulation [7].

Dysregulation of iron metabolism is also implicated in other diseases, for example, type 2 diabetes or obesity [8]. At the same time, patients with these diseases have the highest risk of severe COVID-19, suggesting a possible link between severe COVID-19 and dysregulated iron metabolism.

Therefore, iron chelators, such as deferoxamine, deferiprone and deferasirox, may be a promising therapeutic option for the treatment of severe COVID-19 courses. However, the results of our current study should be extended to other organs in the near future and, because of the small number of patients included in this study, validated in larger patient cohorts. It would be also of interest to study subcellular iron distribution. Since iron serves as a catalyst for the chemical reaction, very small amounts of free iron would be sufficient to trigger ferroptosis [9, 10], even below detection limit of conventional histochemical techniques. Because we used only cardiac tissue samples from deceased patients, interpretation of our results for the living organism, especially for patients with asymptomatic or moderate COVID-19 disease, may be difficult. However, it should be noted that 4-HNE staining correlated significantly with cardiac troponin levels in COVID-19 patients, suggesting that relevant ferroptosis may be present in severe COVID-19 disease. Therefore, the onset and progression of dysregulated iron metabolism during COVID-19 disease should be investigated in further studies.

In conclusion, this study is the first to show a direct association between intracellular iron accumulation, ferroptosis and SARS-CoV-2 infection in the heart during lethal severe disease courses of COVID-19.

## Data Availability

Original data can be made available in a blinded manner upon reasonable request.
